# Artificial Intelligence-Based Grading Quality of Bovine Blastocyst Digital Images: Direct Capture with Juxtaposed Lenses of Smartphone Camera and Stereomicroscope Ocular Lens

**DOI:** 10.3390/s18124440

**Published:** 2018-12-15

**Authors:** Marcelo Fábio Gouveia Nogueira, Vitória Bertogna Guilherme, Micheli Pronunciate, Priscila Helena dos Santos, Diogo Lima Bezerra da Silva, José Celso Rocha

**Affiliations:** 1 Laboratory of Embryonic Micromanipulation, Department of Biological Sciences, School of Sciences and Languages, São Paulo State University (UNESP), Assis, São Paulo 19.806-900, Brazil; marcelo@assis.unesp.br (M.F.G.N.); viihgui@gmail.com (V.B.G.); micheli_prtt@hotmail.com (M.P.); santos.priscilah@gmail.com (P.H.d.S.); 2 Multiuser Facility (FitoFarmaTec), Department of Pharmacology, Biosciences Institute, São Paulo State University (UNESP), Botucatu, São Paulo 18.618-689, Brazil; 3 Laboratory of Applied Mathematics, Department of Biological Sciences, School of Sciences and Languages, São Paulo State University (UNESP), Assis, São Paulo 19.806-900, Brazil; diogolimabsilva@gmail.com

**Keywords:** artificial intelligence, artificial neural networks, embryo grading, bovine blastocyst, image processing, software, digital image capture, smartphone camera

## Abstract

In this study, we developed an online graphical and intuitive interface connected to a server aiming to facilitate professional access worldwide to those facing problems with bovine blastocysts classification. The interface Blasto3Q, where 3Q refers to the three qualities of the blastocyst grading, contains a description of 24 variables that were extracted from the image of the blastocyst and analyzed by three Artificial Neural Networks (ANNs) that classify the same loaded image. The same embryo (i.e., the biological specimen) was submitted to digital image capture by the control group (inverted microscope with 40× magnification) and the experimental group (stereomicroscope with maximum of magnification plus 4× zoom from the cell phone camera). The images obtained from the control and experimental groups were uploaded on Blasto3Q. Each image from both sources was evaluated for segmentation and submitted (only if it could be properly or partially segmented) for automatic quality grade classification by the three ANNs of the Blasto3Q program. Adjustments on the software program through the use of scaling algorithm software were performed to ensure the proper search and segmentation of the embryo in the raw images when they were captured by the smartphone, since this source produced small embryo images compared with those from the inverted microscope. With this new program, 77.8% of the images from smartphones were successfully segmented and from those, 85.7% were evaluated by the Blasto3Q in agreement with the control group.

## 1. Introduction

Brazil is the largest beef exporter in the world with a cattle herd of 218.2 million [1], which is more cattle than the Brazilian population of 207 million in 2017 [2]. Beef production is the major economic activity of the country, accounting for USD $5.3 billion in 2016 and employing 1.6 million people. The industry is also the main producer of in vitro bovine embryos, with 366,517 embryos/year, which is around 70% of the world bovine embryo production [3]. The Brazilian program of in vitro production (IVP) of bovine embryos started in 1990, but the first three births only occurred in 1994 [4]. This achievement used Nellore breed immature oocytes, frozen-thawed semen, and a culture system. Currently, IVP is used commercially for several laboratories to research and multiply genetic material for animal production [5]. This production is of utmost importance for international and national improvement in cattle genetics and productivity.

Animal production has undergone many changes since the 1960s due to the livestock sector and agricultural research. In this context, the development of in vitro techniques for the production of cattle embryos has proven to be useful. The production of bovine embryos for commercial purposes involves the in vitro production, then transferring to synchronized receptors when the embryo reaches the blastocyst stage [6]. For their production, the donor cow undergoes a system called ovum-pick up to retrieve its oocytes (commercial purposes) or the ovarian follicles can be aspirated on ovaries from an abattoir (research purposes) [6].

Compared to the IVP system, there is a more natural way to produce bovine embryos, which is multiple ovulation and embryo transfer (MOET). This treatment increases the number of ovulations to be above the average for the species using hormones and lasting up to 9 or 10 days [6]. Six to eight days after the artificial insemination of the superstimulated MOET cows, the embryo presumptively has reached the uterus. In this stage, the embryos are still surrounded by the zona pellucida and are tolerant to some handling outside of the cow body. Although both in vitro (IVP) and in vivo (MOET) produced embryos are tolerant, the degree of tolerance and their quality are dependent on the original environment (in vivo or in vitro) from where they were derived [6].

There are some differences between embryos derived from IVP or MOET. The embryos produced in vivo are round, brownish, with plasmatic membranes of the blastocysts close to the zona pellucida, and the inner cell mass (ICM) is surrounded by small intercellular spaces [7,8]. The embryos produced in vitro are generally darker (this has been associated with a higher accumulation of lipid or lipid-like granules in the cytoplasm due to the use of serum in the culture medium) [9]. Blastomeres of in vitro embryos appear more swollen, the perivitelline space is smaller at all pre-compaction stages [9], have a decreased cell count [10], and an increased number of vacuoles in comparison to those produced in vivo [11,12].

### 1.1. Classification of Embryos by Morphological Analysis

The embryonic quality is determined based on the number and appearance of cells. This classification follows the International Embryo Transfer Society (IETS) [13], using two codes for description: One to assign the quality and the other to the stage of development. The standard evaluation is performed with a stereomicroscope at 50 to 100× magnification. The diameter of bovine embryo is 120 to 190 µm, including the zona pellucida (12 to 15 µm). The ideal embryo is compact and spherical with blastomeres of similar size, color, and uniform texture; the cytoplasm cannot be granular or vesiculated. The perivitelline space should be clear and the zona pellucida not cracked or collapsed. The code for the stage of development is numeric, varying from “1” for unfertilized oocyte or 1-cell embryo, to “9” for expanding hatched blastocyst. The code for quality is based on the morphological integrity of embryos, ranging from “1” (good or excellent) when symmetrical and spherical, the cellular material is 85% intact and irregularities are relatively minor; to “2” (fair) when the embryos have moderate irregularities in the overall shape of embryonic mass or size and color; “3” (poor) when major irregularities in shape or other parameters are observed; to “4” (to dead or degenerating embryos), which denotes a non-viable structure.

Despite this classification, the evaluation of bovine embryos is directly affected by the embryologist’s accuracy, experience, and mood [14]. The reason for this is that morphological analysis does not measure any objective variables to determine the embryo classification. Human vision is subjectively able to extract information about an image, but some information may be ignored or not observed, and this measurement is often based on a comparison among objects or images. Given the variability in human eyes, when we have to analyze an image, we may experience difficulties in judging color or the brightness of shapes and features [15]. Thus, analyses by an embryologist may have low reproducibility [16]. Farin et al. [17] described two kinds of embryologist error that support this statement. Inter-evaluator error occurs when the same embryo is classified with different quality grades by different embryologists. Intra-evaluator error occurs when the same embryologist classifies the same embryo as different grades. This occurs when the quality grade is borderline, the evaluator is inexperienced, they are tired, or their mood is altered. Due to all these reasons, the embryo evaluation is less reproducible and objective than desired. However, it is one of the most critical steps in the embryo transfer procedure (IVP or MOET) and is strongly related to a successful pregnancy establishment [13].

### 1.2. Semi-Automatized Methods for Evaluating Embryo Quality

Several methods have been or are being developed to improve the process of embryo classification without external effects. Many embryologists select oocytes/embryos using a non-invasive examination based on simple observation focused on morphology and kinetics of the developmental stage (usually on the third day of culture or blastocyst stage) [18]. These methods include embryo metabolism analysis, cellular respiration measurement, evaluation by time-lapse video [19], the quality of in vitro growth of embryos, the integrity of blastomeres membrane [19,20,21], and electron-microscopy analysis [19,22]. Other kinds of evaluation have been used in an attempt to find a better method to classify embryo quality. Melo et al. [23] described an automatic segmentation procedure of bovine embryos without the use of Artificial Intelligence (AI) and the authors calculated the method’s sensitivity, specificity and accuracy. Although the authors obtained positive results with high rates of sensitivity and specificity, the method is not yet applicable to the bovine embryos.

Time-lapse is a non-invasive technology mainly used for human embryos to measure morphokinetic parameters, such as the timing of karyogamy, time intervals between cytokinesis, and its abnormal events that result in uneven blastomere sizes [24,25]. With this technology, the embryos can be monitored without removing them from the incubator as required for other morphological and dynamics analyses. Embryo images are captured by a camera built into the incubator at timed intervals [19,26]. Among the current time-lapse systems, two are the most widely used: Embryoscope/Fertilitech (an incubator with an integrated time-lapse system) [27,28] and Primo Vision/Vitrolife (a compact digital inverted microscope system) [27,28]. Both use bright field technology.

Other methods of embryo quality analysis include the semi-automatized grading method of human blastocysts using a support vector machine (SVM). It performs classification by determining a separation rule between two sets of feature values (support vectors) [29]. Among 17 classification methods tested by Meyer [30], this produced the best performance.

From photographs of oocytes and embryos at the four-cells stage, Manna et al. [18] used techniques based on (1) segmentation (selection of the correct region of interest in the image) and pre-processing for reducing artefacts due to noise, blur, or illumination conditions; (2) feature extraction (usually dependent on numerical descriptors) that compactly represent the starting image (such as local binary patterns or LBP) [18,19,31]; and (3) definition of a classification system in which the classifier is trained using the data stored in the knowledge base.

Several authors proposed the use of mathematical and statistical tools for the evaluation of the embryo quality, such as multivariate logistic regression with eight predictive factors for the classification of embryos according to implantation potential [19,32] and the computer-assisted scoring system (CASS) [19,33] that is supposed to have a higher discriminatory power for embryo selection over the standard scoring system that has intrinsic examiner variability. The multivariate logistic regression (LR) system together with multivariate adaptive regression splines (MARS) was shown to be a better predictive model when using the CASS associated with data mining. Any of these methods are effective for prediction and morphological analysis still is widely used to evaluate embryo quality [19,34].

### 1.3. Artificial Intelligence-Based Techniques

Techniques based on Artificial Intelligence (AI) could potentially support the objective, reproducible, and non-invasive prediction of embryo quality with high levels of accuracy [19]. Among techniques, artificial neural network (ANN) and genetic algorithm (GA) have already been used to simulate an accuracy predictive model [35].

ANNs are distributed parallel systems composed of simple processing units (i.e., the neurons) that compute certain mathematical functions [36,37]. ANN is based on biological neuron functions and how they process the passage of the nerve impulse and consequently learn. What ultimately determines the ANN intelligent behavior is the interactions among the neurons [38].

ANNs can be composed by several layers of interconnected neurons, with an input and an output layer, and some intermediate layers between them (i.e., multilayer networks). An ANN is also composed of the transfer and learning functions that determine how it learns. This learning can occur through supervised and unsupervised algorithms. The set formed by the neurons, their layers, transfer and training functions, and the algorithm of learning is called the ANN architecture [39,40]. This technique has been used for the morphological classification of microscopic images of IVP bovine embryos and produced promising results [41,42].

Associated with ANNs, the GA technique can determine the optimal ANN architecture for a problem under study. The GA involves heuristic searching based on Charles Darwin’s Theory of Natural Selection. The more adapted individuals (i.e., the fittest) survive and pass the features to the offspring, thus leading to the convergence of the features and increasing the adaptability of individuals throughout the generations [43].

To optimize the process that finds the best ANN, the GA technique uses, as the population, a set of ANNs (which are the individuals of the population). This population passes through the processes of recombination, crossover, mutation, and migration. After a pre-established number of generations, the GA provides the best ANN (the fittest) to solve the problem [43].

Found by the GA technique, three ANNs were developed after being trained to evaluate the quality of images from IVP bovine blastocysts based on the International Embryo Technology Society standard [41,42]. In that study, 482 blastocyst images were used to train some ANNs, from which the best obtained a 76.4% accuracy using a fully automatized process.

### 1.4. Blasto3Q Algorithm

The work mentioned above resulted in two different interfaces, one using the MatLab^®^ (MathWorks, Natick, MA, USA) platform and an interface using a multiplatform approach for online purposes. Both were called the “Blasto3Q” program, where 3Q refers to the three qualities of blastocysts evaluation [41,42]. In the first case, the interface (in accordance with the recommendations for commercial use of the program) allows the user to quickly and intuitively interact with the program. This interface contains a description of the 24 variables extracted from the image and analyzed by the ANN that classifies the same loaded image through the best three obtained ANNs. The second developed interface allows the online use of Blasto3Q via a cell phone (smartphone) or PC.

#### 1.4.1. Image Acquisition Process

Using MatLab^®^, it was possible to perform automatized analysis without an embryologist’s intervention. For standardization, the software first performed image import (BMP or JPG), conversion to greyscale (8-bit grayscale), resolution and proportion adjustment (640 × 480 pixels was chosen as the default resolution because it is the lowest standard and provides sufficient information for interpretation), then intensity adjustment (1% of all information becomes saturated between light and dark pixels) [41,42].

#### 1.4.2. Image Segmentation

The segmentation algorithm includes four steps [41,42]. The first step involves calculating the magnitude of the image gradient and the edges are highlighted in all directions. This step is important for the next steps and is essential for characterizing the circular shape of the embryo. Following the magnitude gradient, the binary image is calculated and a value of 128 is selected as the intensity threshold. After that, Circular Hough Transform is used to detect the embryo circumference and map the image to provide an isolated embryo from the background image [42]. The algorithm searches for circles in two stages: In circles with a radius between 100 and 150 pixels and then in circles with a radius between 150 and 200 pixels for greater accuracy. Therefore, both initial blastocysts (smaller) or expanded blastocysts (larger) can be detected. At the end of both searches, in each image, the detected circle’s metrics are compared, and the largest radius is used after the best circle is detected. The last step is blastocyst isolation, where tree versions of the image are generated: (1) The radius of circumference is increased five pixels to ensure that the zona pellucida is included (called the ER); (2) the radius is decreased by 40 pixels to exclude the trophectoderm, selecting just the inner cell mass (ICM) and blastocoel for further analysis (called the RR); and (3) the difference between ER and RR is calculated. By following this method, approximately just the trophectoderm region was isolated in the image (called TE). The pixel values, which determine the expansion (ER) and the contraction (RR) of the blastocysts images, are obtained by assessment of the image database.

The image texture is defined by repeated random regular patterns in a region of the image that provide information on the surface structure [41,44]. It is an important characteristic that is used to identify the regions of interest in an image [45]. The statistical method used to analyze the textures in images was the grey level co-occurrence matrix. Considered the most efficient, it describes the spatial distribution of the pixel value intensity by considering a determined distance and angle, which enables recognizing and classifying textures [46,47].

#### 1.4.3. Extraction of Variables

After image standardization and segmentation, 36 variables were extracted that could represent all the relevant information of the analyzed blastocyst image [41]. Collinearity analysis was performed to eliminate those variables that were considered redundant. Based on the Variance Inflation Factor, collinear variables were considered when the value was higher than 10. Thirteen iterations were performed until all the variables had a value ≤10. Thus, 24 variables remained for use in the ANN.

For the ANN learning process, the backpropagation algorithm was used [34]. The images were divided into training (70%), validation (15%), and test (15%). The accuracy of the obtained ANN was verified according to the error between the real values (embryologists’ evaluation) and the values obtained by the ANN. Currently, there is no standard method for obtaining the best ANN architecture for the fittest solution of a problem [48].

The GA technique developed in that study [48] considered the creation of an initial ANN population with different architectures, which was randomly generated and composed of 100, 200, or 300 individuals (i.e., the ANN architectures). Each one was defined by a “genetic code”, that is, a specific pattern of nine different “genes” representing the variables (e.g., the number of neurons in the first, second, and third hidden layer; the transfer function for the first, second and third hidden layer; the transfer function for the output layer; the training function to be used; and the number of hidden layers to be used). Each ANN was trained and tested, and the embryo grading success was assessed.

The present work is a continuation of previous work [49,50], extending the model with new data and aiming at the development of a Graphical User Interface for users that cannot purchase or access an inverted microscope to capture digital images with high magnification. In addition, embryologists from around the word could access the technique online, without downloading or installing executable software, and view an image captured by the cell phone camera.

## 2. Materials and Methods

Blastocyst images were captured by two datasets (1 and 2). Dataset 1 was derived from two biological replicates and 18 embryos were used for image capture both from an inverted microscope (control group) and a smartphone source (experimental groups I and II, respectively). Smartphone images in this dataset produced a bigger area and interception of the proper embryo in the total area of the image compared with smartphone images from Dataset 2 (experimental group III). This set was obtained with three biological replicates (*n* = 25 embryos) and the inverted microscope was not used; only images from the smartphone were captured.

Embryos were produced in vitro from the cumulus-oocytes complex from the antral follicles of slaughtered cows based on the protocol previously published [51]. On days 7 to 8.5 of in vitro culture (day 0 was in vitro insemination), the embryos were recovered from the culture. Only the embryos morphologically classified as at a minimum stage of blastocyst or with inner cell mass (ICM) relatively smaller than blastocoel and a thin layer of trophectoderm [13] were evaluated. First for Dataset 1, the blastocysts were separated on phosphate buffered saline (PBS) solution (Nutricell, Campinas, São Paulo, Brazil) for image capture. For the first experimental group, the embryo digital image was captured with a smartphone G4 Plus (Motorola, Chicago, IL, USA) and ANDROID™ 7.0 system (Google, Mountain View, CA, USA) with 16 megapixels of camera, lens aperture f/2.0 and digital zoom of 4× through the ocular lens from a stereomicroscope S8 APO, 10× magnification (Leica, Wetzlar, Germany) with maximum zoom magnification. The files were generated in JPG format. Different from a previous study [49], it was not necessary the use an extra macro lens coupled to the smartphone by a clip to capture the images from the stereomicroscope. In this case, only the smartphone zoom was used to capture images.

**Figure 1:**
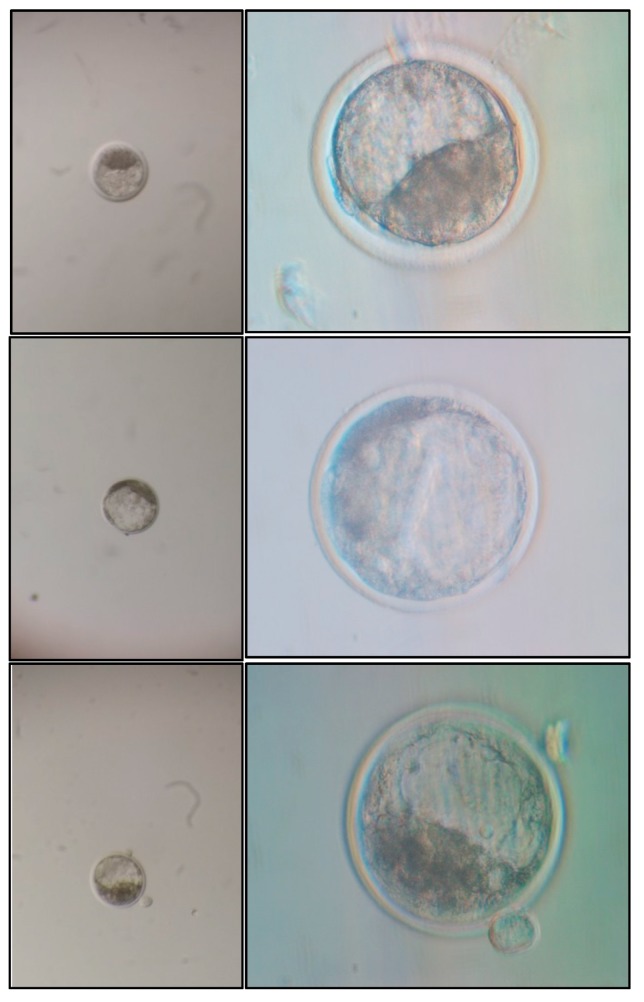
Illustrative images captured by the smartphone without a macro lens (**left** column) and with an inverted microscope (**right** column), from three different embryos (**upper**, **middle**, and **bottom** lines).

The images of the same embryos were also acquired by an inverted microscope Eclipse T*i* (Nikon, Tokyo, Japan) coupled to a Digital Sight DSRi1 (Nikon, Tokyo, Japan) at 40× magnification in the Nikon software NIS Elements Ar 3.0 (control group). The images were stored in JPG format in 8-bit color (RGB) at a resolution of 1280 × 1024 pixels. To capture pictures with the smartphone and inverted microscope, the embryos were adjusted in a standardized position with ICM perpendicular to the focal plane (Figure 1). Each picture contained only one blastocyst. Notably, when images were obtained with the smartphone, the embryo occupied a smaller area of the entire image compared with images from the inverted microscope (Figure 1).

Using the ImageJ program [52] the embryos from smartphone images (Groups I and II; Dataset 1) occupied on average 3.01% of the total area of the image and their diameter was intercepted, on average, by 21.57% of a line from the extreme points at the largest width of the image. In the control group, the embryos had an average occupation of 26.95% of the total area and they were intercepted on average by 49.61% of the maximum width.

In Group III (Dataset 2), the digital images were captured with a Motorola smartphone G4 (ANDROID™ 7.0 system) with 13 megapixels of camera, lens aperture of f/2.0 and digital zoom of 4× and without an extra macro lens. This dataset was also analyzed utilizing ImageJ and the embryos occupied on average 0.24% of the total area of the image and their diameter was intercepted, on average, by 4.64% of the line from the extreme points at the largest width of the image. This second set of images was considered the most difficult for the processing and segmentation steps.

### 2.1. Smartphone Image Resizing

**Figure 2:**
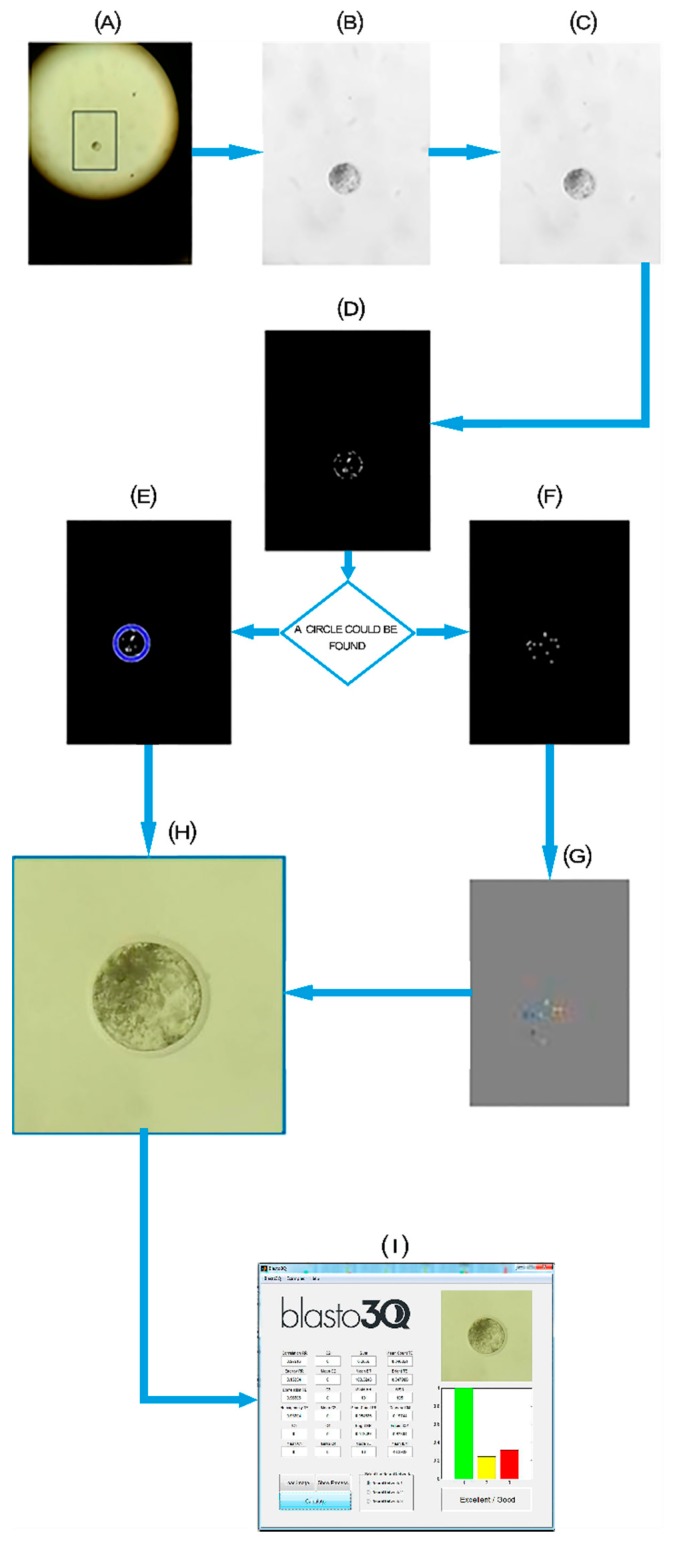
Flowchart of the scaling algorithm software processing steps. (**A**) Originally obtained image with the embryo inside the box; (**B**) conversion of image (A) to grayscale; (**C**) adjustment of the image intensity values; (**D**) binary image; (**E**) Circular Hough transform; (**F**) image obtained with distance transform; (**G**) Watershed transform; (**H**) re-scaled image (expanded); and (**I**) user interface of the Blasto3Q software after expanded image grading.

After a pilot experiment where we tested the original image processing that was previously described [41], we observed poor image processing [50]. Thus, to improve the image processing from the smartphone source to be feasible for use in Blasto3Q software, we processed the images by cutting and enlarging the images with a scaling algorithm software. The software first imports the images from the smartphone source, then performs histogram equalization to correct the brightness of the image and converts it to grayscale (Figure 2). The image then has its contrast magnified and is converted into binary. After this, the algorithm identifies the blastocyst using the circular Hough transform with a variable analyzed pixel range. If the embryo cannot be found by the Hough transform, the algorithm uses the Watershed transform to try to identify the region of the image that includes the blastocyst. After identification of the blastocyst, the image is cut off using it as a reference and then expanded using the bicubic interpolation shown in Equation (1) to maintain as much information of the original image as possible (Figure 2).

(1) $$f\left(x,y\right)=\sum_{i=0}^{3}\sum_{j=0}^{3}a_{ij}x^{i}y^{j},$$

where $a_{ij}$ is the coefficient to be determined and (*x*, *y*) are the points to be interpolated.

### 2.2. Smartphone Image Analysis by Blasto3Q

**Figure 3:**
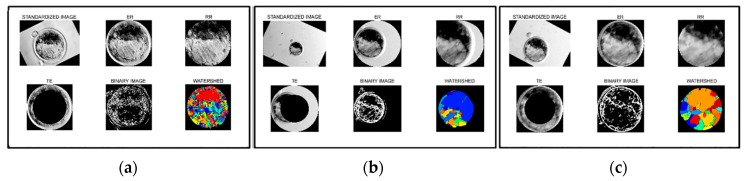
Illustrative comparison among the segmentation of the images from the same embryo but captured by: (**a**) An inverted microscope (control group; Dataset 1), (**b**) a smartphone and without the use of the scaling algorithm software (experimental group I; Dataset 1), and (**c**) a smartphone and after application of the scaling algorithm (experimental group II; Dataset 1). Full (**a**,**c**) and partial segmentation (**b**) were observed depending on the source of the image (**a**,**b**) and the use of scaling algorithm (**b**,**c**).

To analyze the acquired images, we used the desktop version of Blasto3Q software as previously described [41,42]. First, the images were uploaded on the program. In the software, the images underwent an automatized segmentation process, whereby the images were properly isolated from the background. After finishing the segmentation process, the numerical variables were extracted for a classification by the three trained algorithms of Blasto3Q. The images were separated by their degree of segmentation as full (Figure 3a,c), partial (Figure 3b), or non-segmented.

**Figure 4:**
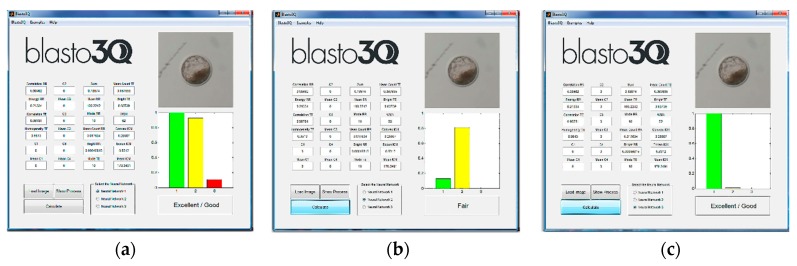
Different graphic interfaces after analyzing the same uploaded blastocyst image from a smartphone source. The mode of the results graded the embryo as (**a**,**c**) “excellent/good”, although (**b**) one Artificial Neural Network (ANN) produced an alternative result (“fair”).

After, the images were submitted for quality grade classification by the three ANNs of the program. For this, each of the ANNs are independent and the program provides a graphic interface to analyze the uploaded image. The user can choose one of the provided results or choose the mode among them (Figure 4). In this work, we used the most prevalent grade (i.e., the mode) provided by the three ANNs from the same uploaded image.

The observed frequencies related with segmentation and grading agreement to the control were statistically compared among (Chi-square) or between experimental groups (Fisher Exact Test). The software SigmaStat (Systat Software, San Jose, CA, USA) was used for the tests and a level of significance of 5% was considered.

## 3. Results

Eighteen in vitro-produced blastocysts were used to obtain distinct images in Dataset 1 where two biological replicates were performed with 11 and 7 embryos. Digital images were captured of the same embryo in the control group (inverted microscope with 40× of magnification), experimental group I (stereomicroscope with maximum of magnification 80× plus 4× zoom from the smartphone) and experimental group II (smartphone image after application of the scaling algorithm software). A total of 36 paired images obtained from control and experimental groups were uploaded on the program Blasto3Q in the form of an executable file.

Each image from three sources was evaluated for segmentation (i.e., the proper isolation of the embryo and of its parts), since that isolation is a prerequisite for the program to obtain numerical values from segmented parts of the image. All images from the control group were properly segmented (Figure 3a). In experimental Group I, 38.9% of the images (7/18) could not be segmented at all, thus no numerical variables were extracted from the images and no quality grade for the embryo could be evaluated by Blasto3Q. In this group, 11 images (61.1%; 11/18) were partially segmented, meaning that although the embryo was isolated and numerical values could be extracted, there was a mismatch and some parts of the embryo were lost during the segmentation steps (e.g., Figure 3b). In the experimental Group II, 22.2% of the images (4/18) could not be segmented at all. However, the remaining images (77.8%; 14/18) were fully segmented (e.g., Figure 3c).

**Figure 5:**
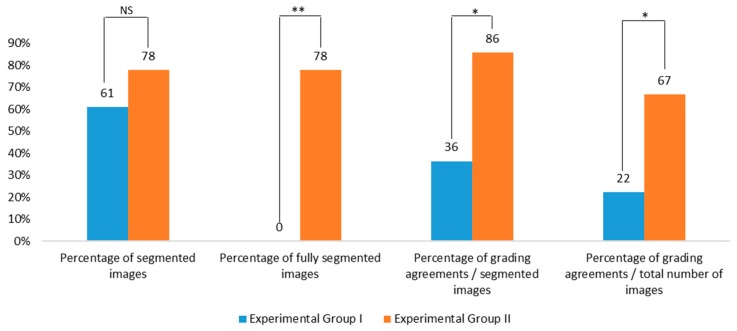
Results of segmentation and grading agreement (related to the grading image produced by control group) of experimental groups I and II (Dataset 1). Fisher Exact Test: NS: *p* = 0.471), ** *p* < 0.001, and * *p* < 0.02.

The images, properly (control group and experimental Group II) or partially segmented (experimental Group I), were submitted for grading by the three ANNs of the Blasto3Q program. Considering the mode of the classification for each image and the control group as the standard, there was an agreement of 36.4% (4/11) and 85.7% (12/14) for groups I and II (Figure 5), respectively. The increase could be related to the use of the scaling algorithm, since the raw images were the same. Moreover, there was a statistically significant increase in grading agreement when all images (segmented or not) where compared between Groups I and II (Figure 5).

**Figure 6:**
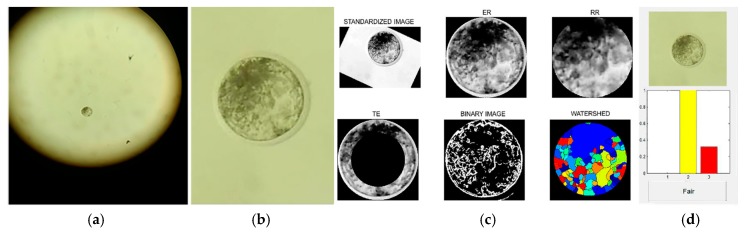
(**a**) Original image from the smartphone (Dataset 2). (**b**) Image expanded by resizing (scaling algorithm software). (**c**) Blastocyst segmentation by Blasto3Q. (**d**) Final output (blastocyst classification as “fair”) of Blasto3Q.

In Group III (Dataset 2), the captured embryo images (*n* = 25) were submitted to the scaling algorithm, where 44.0% (11/25) were correctly segmented and the remaining 14 images were not. After image resizing, they were uploaded to the Blasto3Q algorithm (Figure 6) and a grading value was produced as expected.

When both datasets were compared, even with the use of the scaling algorithm software for the smallest embryo in the images from Dataset 2, the same segmentation performance observed with images from Dataset 1 could not be produced (Table 1).

## 4. Discussion

The alternative source (smartphone) for image capture produced an embryo characterization that could prevent the Blasto3Q program from properly classifying it. This occurred when the program adjusted to process the image captured from an inverted microscope was also used for the smartphone-captured images. In general, the latter were images with proportionally smaller embryos. This was the main cause of the impaired image processing observed with the Blasto3Q program once it was trained with the higher percentage of area and interception of the embryo image covering the total area of the picture when the source was an inverted microscope. Consequently, the original program was able to properly search and process the segmentation steps with a higher embryo diameter in the uploaded image. This was proved by a pilot study [50] where we observed the total failure of 18 smartphone images to be properly segmented by Blasto3Q in its original configuration.

As ANN is based on the machine learning concept, the Blasto3Q algorithm learned to classify blastocysts from the pattern observed by an inverted microscope capture. As such, its training was lost because the evaluation is based on the numerical values extracted from the segmented image [41]. As described, no image could be fully segmented and only 61.1% were partially segmented in experimental Group I. The numerical variables extracted from partially segmentation produced a mismatch with the real information contained in the full embryo image. Thus, the bad quality variables extracted (experimental Group I) were unable to match the quality evaluation from the control group, with only a 36.4% classification agreement between them.

As hypothesized [50], a simple readjustment in the radius size when searching for the embryo (originally adjusted for a higher radius in Blasto3Q, since it was based entirely on inverted microscope images) improved the results of segmentation and grading. The radius size is a parameter used by Hough transform to find a circularity in the total image, providing a process to segment only the embryo by its almost perfect and constant circularity.

It was not necessary to examine the other inferred possibility to correct the error [50], which is the de novo training of the algorithm with a full database composed only of smartphone blastocyst pictures. Avoiding this alternative allowed the original training of the program to continue properly evaluating inverted microscope images, and, after the use of scaling algorithm software, to grade images from the smartphone camera. As far as we know, there has been no similar attempt (published or as communication) like that presented here. As such, it is difficult to compare our results with others. In summary, our concept involves blastocyst images (captured solely by a smartphone lens and a stereomicroscope ocular lens) to be automatized evaluated by software based on ANN and trained by larger images from an inverted microscope. No executable file is necessary, just a mobile app, and both sources of images, both large or small depending how it was captured), could be graded.

As smartphones are being constantly technically improved, their use due to their resources (good quality camera lens, applicative to image capture and zoom, and easy connection to the internet to send and receive data) is increasingly supporting approaches based on AI techniques to classify digital images [53] or simply perform an analysis without AI-based techniques [54]. Thus, this work is in accordance with this new trend of portable computerized systems performing automatized and objective analyses.

## 5. Conclusions

With improvements in the image processing steps (i.e., the scaling algorithm software), a blastocyst image directly captured from a stereomicroscope ocular lens could be evaluated properly by the Blasto3Q program. It was not necessary to use a macro lens with the smartphone lens to capture the images. The size (area and percentage of interception) of the embryo in the captured image from the smartphone had a technical impairment. The processing of the smallest embryo in the image (Dataset 2) was not the best result obtained with the biggest embryo (Dataset 1). Even so, the 67% overall agreement (Dataset 1, when considering all images) requires improvements.

## 6. Patents

Patent number PCT/BR2013/000506 (WIPO, 19.06.2014) covers the Blasto3Q program itself and its graphic interface.

## Figures and Tables

**Table 1:** Percentage of segmentation among experimental groups. Larger (Groups I and II) and smaller images (Group III) captured from a smartphone source were segmented with (Groups II and III) or without (Group I) the use of the scaling algorithm software. The observed frequency among the three groups were compared with Chi-square, whereas Groups II and III (fully segmented images) with Fisher Exact Test.

	Dataset 1		Dataset 2
	*n* = 18		*n* = 25
**Experimental group**	I	II	III
**Total segmented images (%)**	11 (61.1)	14 (77.8)	11 (44.0) *
**Fully segmented images (%)**	0 (0.0)	14 (77.8 ^a^)	11 (44.0 ^b^) **

*, ** Chi-square among the three groups, respectively *p* = 0.083 and *p* < 0.001 for * and **. ^a,b^ Fisher Exact Test between groups II and III (*p* = 0.034).
